# Comparative study on the toxic mechanisms of medical nanosilver and silver ions on the antioxidant system of erythrocytes: from the aspects of antioxidant enzyme activities and molecular interaction mechanisms

**DOI:** 10.1186/s12951-019-0502-2

**Published:** 2019-05-17

**Authors:** Wenxu Fang, Zhenxing Chi, Weiguo Li, Xunuo Zhang, Qiang Zhang

**Affiliations:** 1 Department of Environmental Engineering, Harbin Institute of Technology, Weihai, 2# Wenhua West Road, Weihai, 264209 People’s Republic of China; 2 Guangzhou Key Laboratory of Environmental Exposure and Health, School of Environment, Jinan University, Guangzhou, 510632 People’s Republic of China; 3 State Key Laboratory of Urban Water Resource and Environment, School of Environment, Harbin Institute of Technology, Harbin, 150090 People’s Republic of China

**Keywords:** Silver nanoparticles, Silver ions, Toxicity, Red blood cells, Antioxidant enzyme

## Abstract

**Background:**

The wide application of silver nanoparticles (AgNPs) in medicals and daily utensils increases the risk of human exposure. The study on cell and protein changes induced by medical AgNPs (20 nm) and Ag^+^ gave insights into the toxicity mechanisms of them.

**Results:**

AgNPs and Ag^+^ affected the enzymatic and non-enzymatic antioxidant systems of red blood cells (RBCs). When RBCs were exposed to AgNPs or Ag^+^ (0–0.24 μg/mL), catalase (CAT), superoxide dismutase (SOD) and glutathione peroxidase (GPX) were more sensitive to Ag^+^, whereas the RBCs had slightly higher glutathione (GSH) contents treated by AgNPs. Both AgNPs and Ag^+^ increased the malondialdehyde (MDA) content of RBCs, but the difference was not significant. The difference in the change of the enzyme activity indicated that AgNPs and Ag^+^ have different influencing mechanisms on CAT and GPX. And SOD has stronger resistance to both of AgNPs and Ag^+^. When AgNPs or Ag^+^ (0–10 μg/mL) was directly applied on enzymatic proteins, although AgNPs or Ag^+^ at a high concentration was toxic, at the concentration below 0.4 μg/mL could promote the activities of CAT/SOD/GPX. The spectroscopic results (fluorescence, synchronous fluorescence, resonance light scattering and ultraviolet absorption), including the changes in amino acid microenvironment, peptide chain conformation, and aggregation state, indicated that the interaction mechanism and conformational changes were also the important factors for the changes in the activities of SOD/CAT when SOD/CAT were directly exposed to AgNPs or Ag^+^.

**Conclusions:**

Low concentration (< 0.4 μg/mL) of AgNPs is relatively safe and the direct effects of AgNPs and Ag^+^ on enzymes are important reasons for the change in antioxidant capacity of RBCs. 
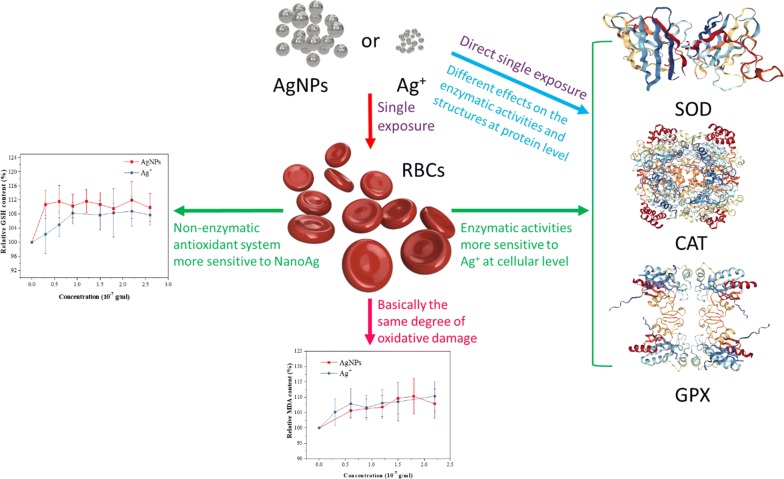

## Introduction

With the development of nanotechnology, nanosilver has been widely applied in industrial and biomedical products. Nanosilver is important for transparent conductive coatings in fabricating printed electronic devices such as flexible displays and solar cells due to its excellent conductivity and thermal conductivity [1]. The antibacterial activity of AgNPs is well known [2]. Therefore, AgNPs are applied in household appliances, toothpaste, and medical bandages to achieve antibacterial and anti-infection effects [3]. However, these applications resulted in the accumulation of AgNPs in the environment and organisms [4–9]. Due to these applications, residues and bioaccumulation of AgNPs have increased their exposure to human beings, thereby increasing the potential risks of toxicity [10].

AgNPs toxicity affected all kind of cells from bacteria to eukaryotes and even viruses [11]. AgNPs of certain concentrations about over 10 μg/mL have significant toxic effects on the proliferation of mammalian cells, such as human blood mononuclear cells [12], mesenchymal stem cells [13], human bronchial epithelial (BEAS-2B) cells [14], mouse vascular endothelial cells [15], spermatogonial stem cells [16], mouse embryonic fibroblast cell line and human breast carcinoma cell line [17]. AgNPs are also toxic to many species including HIV-1 [18], fish [19–21], rabbit [22], halophilic microalgae [23], green freshwater algae [24], and *Arabidopsis* [25].

AgNPs themselves were toxic because of their size and shape or released silver ions, which are well known for their antibacterial and other destructive behaviors [26]. Durán et al. [27] concluded that the toxicity of AgNPs had three possible toxicity mechanisms. Firstly, free Ag^+^ uptaken by cells destroy the production of ATP and DNA replication. Secondly, the initiation of reactive oxygen species (ROS) is promoted on the surface of AgNPs and Ag ions. Thirdly, AgNPs directly damage cell membrane. The comparative study [9] on the toxicity of AgNPs and Ag^+^ to *Escherichia coli* suggested that the release of bioavailable Ag ions from AgNPs promoted a higher toxicity. In addition, 10 μg/mL AgNPs treatment had toxic effects on the early growth of wheat seedlings and the AgNPs effects observed were primarily ascribed to Ag ions released by oxidative dissolution at the root interface in the presence of secreted root metabolites [28]. The marine microalgae *D. salina* were also found to be more sensitive to Ag ions than AgNPs [23]. However, Greulich et al. [29] reported that the effective toxic concentrations of AgNPs and Ag^+^ towards bacteria and human cells were almost the same.

Many toxic effects of AgNPs on RBCs (from common carp [30], *Mus musculus* [31], rats [32, 33], and human [34]) have been reported. The cytotoxicity of AgNPs on RBCs was ascribed [35] to the direct interaction between nanoparticles and RBCs, which resulted in the production of oxidative stress, membrane injury, and subsequent hemolysis. However, the comparative study on the toxicity of silver nanoparticles and silver ions to RBCs was seldom reported.

As the main function, oxygen transport causes RBCs to be exposed to a significant dose of continuous oxidative stress [36]. Therefore, antioxidants systems are important for the RBCs. Yet the toxicity mechanism of AgNPs is unclear and whether AgNPs are more toxic than Ag^+^ to the antioxidant capacity of human red blood cells should be further confirmed. This study explores the effects of AgNPs and Ag^+^ on the antioxidant capacity of red blood cells and the activities and structures of several key antioxidant enzymes. The study gives insights into the toxicity mechanism of AgNPs and Ag^+^ and provides basic data for their application and environmental protection.

## Materials and methods

### Reagents and apparatus

EDTA-K_2_ (Tianjin Kermel Chemical Reagent Co., Ltd.) stabilized blood samples were obtained from the Weihai Blood Centre in China (Ethics statement: The study was approved by the Ethics Committee of Weihai Blood Centre). AgNPs with an average diameter of 20 nm was purchased from Nanjing/Jiangsu XFNANO Materials Tech Co., Ltd. (NH_4_)_2_SO_4_, NaOH, NaCl and AgNO_3_ was purchased from Sinopharm Chemical Reagent Co., Ltd. The pH of phosphate buffer saline (PBS) is 7.4. The silver solutions were preserved in the dark at room temperature and diluted to different concentrations as required. PBS, SOD (Cu/Zn-SOD, from bovine RBCs) and CAT (from bovine liver) were purchased from Beijing Biodee Biotechnology Co., Ltd. GPX was extracted from human RBCs [37] with ammonium sulfate precipitation method and purified by ion exchange chromatography with DEAE cellulose (DEAE-Crystarose Fast Flow, Wuhan Jing Cheng Bio Technology Co. Ltd.). Ultrafiltration centrifugal tubes (15KD, Millipore) were used to concentrate GPX and desalination. Bicinchoninic acid protein assay kit and GPX activity kit (Nanjing Jiancheng Bioengineering Institute) were used to detect the concentration and activity of purified GPX.

UV–visible absorption spectra were measured with a U-2910 spectrophotometer (Hitachi, Japan). Centrifugation of samples was performed with a CR21N High-Speed Refrigerated Centrifuge (Hitachi, Japan). A digital dry bath incubator (HB-100, Hangzhou Bioer Technology Co., Ltd) was used to control the temperature. Fluorescence measurements were performed with a F2700 fluorescence spectrometer (Hitachi, Japan).

### Cytotoxicity of AgNPs and Ag^+^ to RBCs

The activities or contents of typical biomolecules were determined according to our previous methods [38]. In brief, the fresh blood sample was washed, diluted and mixed with different concentrations of AgNPs or Ag^+^. After incubated at 37 °C for 2 h, PBS in the mixed solution was replaced by ultrapure water to achieve hemolysis. The hemolytic blood samples were used to measure the antioxidant defence capacity of RBCs by the detection kits (Nanjing Jiancheng Bioengineering Institute), namely, the relative activities or contents of CAT, SOD, GPX, GSH [2-nitrobenzoic acid (DTNB) method] and MDA [thio-barbituric acid (TBA) method]. The experimental data were expressed as mean ± SD (standard deviation).

### Effects of AgNPs and Ag^+^ on the activity of CAT/SOD/GPX at molecular level

A certain amount of PBS buffer solution (pH 7.4), CAT/SOD/GPX storage liquid, different amounts of ultrapure water, and AgNPs or Ag^+^ solution were added into tubes. After mixing, the solutions were kept away from light for 30 min at room temperature. After the reaction was completed, the enzyme activity in each tube was determined by corresponding kits. The brief descriptions of the methods are as follows: (1) Superoxide radical anion (${\text{O}}_{2}^{ - } \cdot$) generated from the xanthine and xanthine oxidase system can oxidize hydroxylamine to nitrite which causes absorbance at 550 nm. The SOD in the samples can catalyse the ${\text{O}}_{2}^{ - } \cdot$ and lead to the reduction of nitrite, therefore reduced the colorimetric signal. (2) CAT can catalyse H_2_O_2_ into H_2_O and O_2_, which could be terminated by adding ammonium molybdate. Then the rest of H_2_O_2_ and ammonium molybdate produced a yellow complex which could be measured at 405 nm. (3) GPX can catalyse the reaction of H_2_O_2_ with GSH to H_2_O and oxidized glutathione (GSSG). The activity of GPX was determined by measuring the consumption of GSH in the reaction.

### Effects of AgNPs and Ag^+^ on the molecular structure of CAT/SOD

#### Fluorescence measurements and synchronous fluorescence

The excitation wavelength (λ_ex_) range, the emission wavelength (λ_em_) range, the concentrations of CAT/SOD and AgNPs/Ag^+^ and pH are shown in Figs. 3, 4 and 5. The slit width was 5 nm and the voltage was 400 V.

#### Resonance light scattering measurements

Resonance light scattering (RLS) was measured at λ_ex_ = λ_em_ from 220 to 700 nm. The RLS spectra of CAT/SOD–AgNPs, CAT/SOD–Ag^+^, CAT/SOD, AgNPs and Ag^+^ solution were measured to identify whether the AgNPs/Ag^+^ and CAT/SOD molecules were well mixed in the solution [39].

#### UV–visible absorption spectra

The UV–visible absorption spectra in the range of 190–300 nm was determined with the samples in 1.0 cm × 1.0 cm quartz cuvettes (T = 298 K and pH 7.4).

## Results and discussion

### Effects of AgNPs and Ag^+^ on typical biomolecules of RBCs

**Fig. 1:**
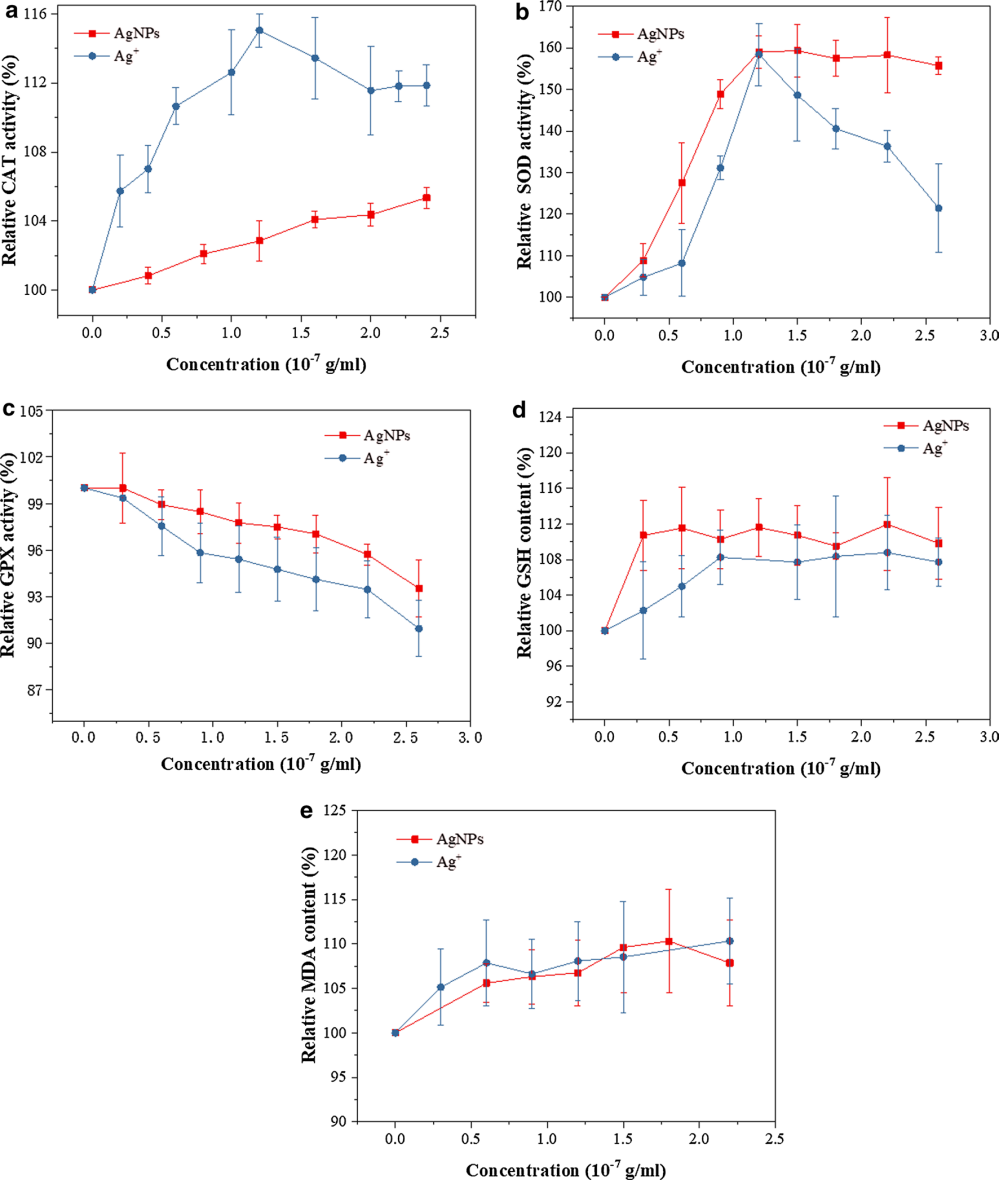
The change of relative activities or contents of CAT (**a**), SOD (**b**), GPX (**c**), GSH (**d**) and MDA (**e**) of RBCs with increasing the concentrations of AgNPs and Ag^+^

The enzymatic antioxidant system (CAT, SOD and GPX) of RBCs is important for cellular biochemical functionality. The balance of these three enzymes is related with the antioxidant function [40]. Figure 1a–c show the effects of AgNPs and Ag^+^ on the relative activities of CAT, SOD and GPX, respectively. When Ag^+^ concentration increased from 0 to 1.2 × 10^−7^ g/mL, the relative activities of CAT and SOD increased to 115% and 158%, thus enhancing the degradation of hydrogen peroxide [41] and the removal of superoxide anion radical [42]. When Ag^+^ concentration further increased to 2.6 × 10^−7^ g/mL, the relative activities of CAT and SOD decreased may due to the binding of metal ions with biomolecules [43], but the relative activities of CAT and SOD were still greater than 100%. With the increase in AgNPs concentration, the relative activity of CAT increased gradually, whereas the relative activity of SOD largely increased to 160% and was then maintained at the high level. When AgNPs or Ag^+^ concentration increased from 0 to 2.4 × 10^−7^ g/mL, the relative activities of GPX decreased to 94% and 91%, respectively. The decrease in GPX activity reduced the reaction of GSH with hydrogen peroxide [44].

The non-enzymatic antioxidant system also contributes to the defence of oxidative damage of cells. GSH is a typical non-enzymatic antioxidant maintaining the redox balance of RBCs [45, 46]. Figure 1d shows that both AgNPs and Ag^+^ affect the contents of GSH of RBCs. With the increase in the concentrations of AgNPs and Ag^+^, the contents of GSH in RBCs firstly increased and then became relatively stable. The content of GSH in RBCs treated with AgNPs was higher than that in RBCs treated with Ag^+^. The MDA is produced from lipid peroxidation after oxidative injury and its content indicates the oxidative stress level of RBCs [47]. As shown in Fig. 1e, in the concentration range of 0.3 × 10^−7^ to 2.6 × 10^−7^ g/mL, both AgNPs and Ag^+^ can cause the oxidative injury and increase the relative MDA content, but the difference is not significant.

Overall, the effects of AgNPs and Ag^+^ on the activities of antioxidant enzymes or the content of GSH were different. However, both AgNPs and Ag^+^ could result in oxidative injury (increased MDA content) to the similar degree.

### Direct effects of AgNPs and Ag^+^ on the activities of CAT/SOD/GPX

**Fig. 2:**
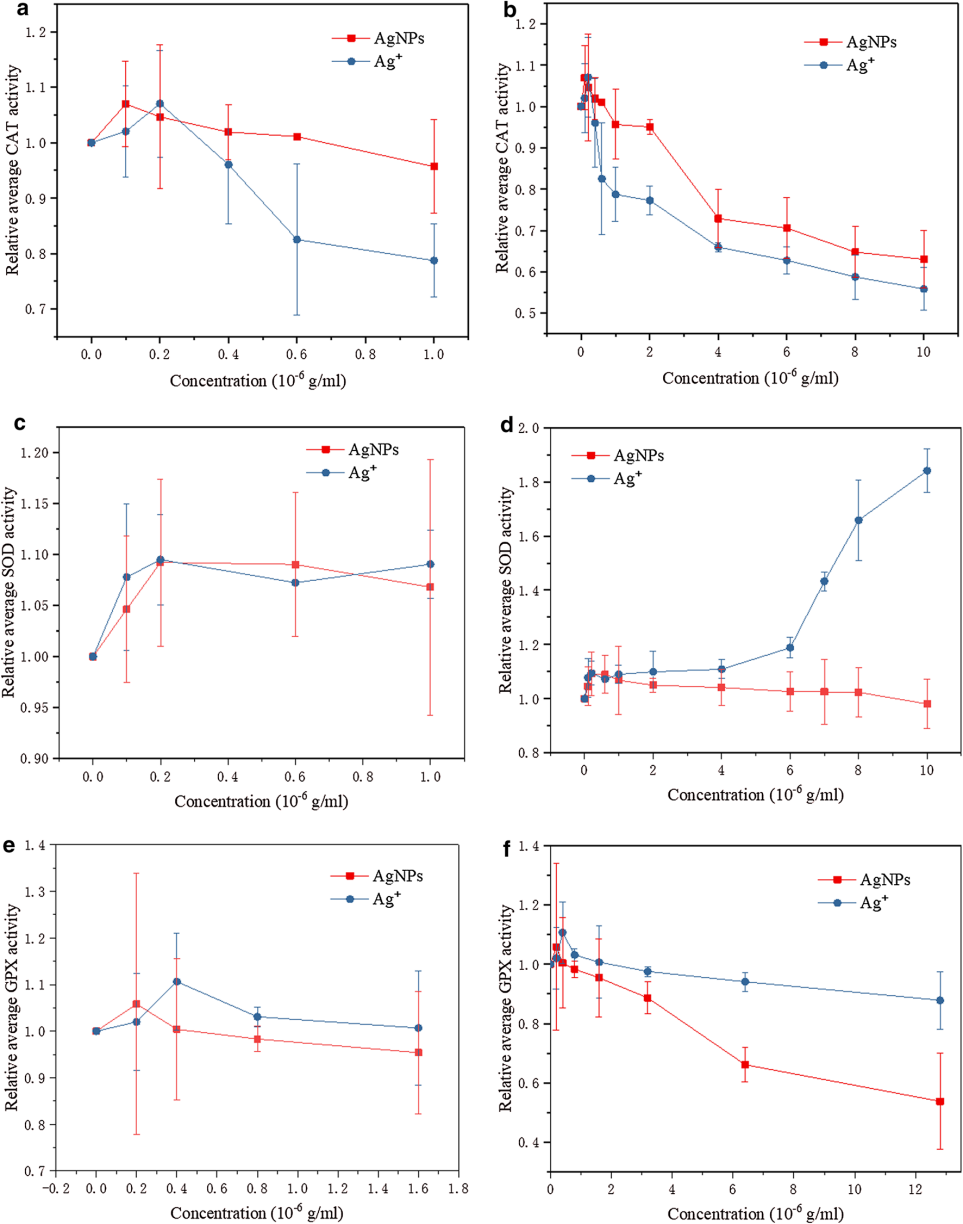
The activity of CAT (**a**, **b**), SOD (**c**, **d**) and GPX (**e**, **f**) in the presence of AgNPs or Ag^+^ at different concentrations. Condition: pH 7.4, T = 298 K, C(CAT) = 2 × 10^−7^ mol/L, C(SOD) = 2 × 10^−7^ mol/L, C(GPX) = 1.6 × 10^−6^ mol/L

The effects of AgNPs or Ag^+^ on the activities of the antioxidant enzymes may rely on the direct action of AgNPs or Ag^+^ on protein molecules. The effects of different doses of AgNPs or Ag^+^ on the activities of antioxidant enzymes in vitro were investigated to validate above interpretation on the effects of AgNPs or Ag^+^. The experimental results are illustrated in Fig. 2.

Under the low concentration of AgNPs (0–1.0 × 10^−7^ g/mL) or Ag^+^ (0–2.0 × 10^−7^ g/mL), which is similar to the cell exposure level, the relative activity of CAT was increased by 7% with the increase in AgNPs or Ag^+^ concentration (Fig. 2a, b). The results were consistent with the results in the exposure at cell level. With the continuous increase in AgNPs concentration (1.0 × 10^−7^ g/mL to 2.0 × 10^−7^ g/mL), the relative activity of CAT began to decrease, but it was still greater than 100%. With the continuous increase in AgNPs concentration (from 6.0 × 10^−7^ to 1.0 × 10^−5^ g/mL) or Ag^+^ concentration (from 2.0 × 10^−7^ to 1.0 × 10^−5^ g/mL), the activity of CAT began to be inhibited and the relative activity finally decreased to 63% and 56%, respectively. The degree of the decline of CAT activity caused by Ag^+^ was larger than that caused by AgNPs.

When the concentration of AgNPs or Ag^+^ increased under the low concentration (0–1.0 × 10^−6^ g/mL), the relative activity of SOD increased to about 110% and the degree of variability was the same (Fig. 2c, d). The results were consistent with the results in the exposure at cell level. SOD activity was not inhibited until the concentration of AgNPs was increased above 8.0 × 10^−6^ g/mL. As the concentration increased (from 1.0 × 10^−6^ to 1.0 × 10^−5^ g/mL), Ag^+^ continued to promote SOD relative activity from 109 to 184%, whereas AgNPs promoted SOD relative activity to decrease from 107 to 98%. Under the higher Ag^+^ concentration (from 1.0 × 10^−6^ to 1.0 × 10^−5^ g/mL), Ag^+^ had the greater influence on SOD activity than AgNPs. The results were opposite to the results in the exposure at cell level. The causes for the opposite results remained to be further explored. We speculate that the reason for the opposite results may be that cell membranes has a protective effect for cell-level exposure compared with direct enzyme exposure, and AgNPs have different with Ag^+^ in the penetration through cell membranes. However, under the similar concentration of AgNPs or Ag^+^, the changing trend of SOD relative activity at enzyme level was similar to that at cell level.

Compared with Ag^+^, AgNPs had the stronger effect on GPX activity (Fig. 2e, f). When AgNPs concentration was greater than 8.0 × 10^−7^ g/mL, GPX activity began to be inhibited. When the Ag^+^ concentration was greater than 1.6 × 10^−6^ g/mL, GPX activity began to be inhibited. When the concentration of AgNPs increased from 8.0 × 10^−7^ to 12.8 × 10^−6^ g/mL, the GPX relative activity decreased from 98 to 54%. When the concentration of Ag^+^ increased from 1.6 × 10^−6^ to 12.8 × 10^−6^ g/mL, the GPX relative activity decreased from 100 to 88%. With the increase in the concentration of Ag^+^ or AgNPs, the decreasing degree of GPX activity caused by AgNPs was significantly greater than that caused by Ag^+^. We speculated that the direct interaction of AgNPs or Ag^+^ with GPX might lead to the change in its activity, which was one of the reasons for the decrease in erythrocyte GPX activity caused by AgNPs and Ag^+^.

In brief, the influences of AgNPs on the activities of CAT, SOD and GPX in vitro were different from those of Ag^+^ on their activities. The difference in the cell membrane permeation ability of AgNPs and Ag^+^ may led to the difference in their effects on the enzyme activities of RBCs. However, under similar exposure concentrations, the influences of AgNPs or Ag^+^ on the CAT, SOD and GPX activities at cellular and molecular levels showed certain similarity. We deduced that the direct interaction between molecules (AgNPs/Ag^+^ and CAT/SOD/GPX) was one of the important reasons for the change in the antioxidant enzyme activity of RBCs.

### Influences of AgNPs and Ag^+^ on the fluorescence intensity of CAT/SOD

In general, most proteins [48–50] have the intrinsic fluorescence due to tryptophan (Trp), tyrosine (Tyr) and phenylalanine (Phe) residues. The intrinsic fluorescence of most proteins is mainly ascribed to Trp and Tyr residues since Phe residue has a low quantum [51, 52]. The fluorescence quenching of protein caused by certain particles can reflect the degree of binding and binding mechanism between these particles and proteins [53]. The fluorescence quenching of SOD [54] and CAT [55] caused by the increase in AgNPs concentration should be ascribed to dynamic quenching. Ag^+^ was also a strong quencher of ovalbumin fluorescence and the complexes of Ag^+^ with sulfhydryl compounds also generated mercaptide absorption bands capable of quenching indole fluorescence by the energy transfer mechanism [56]. The fluorescence quenching of bovine serum albumin by Ag^+^ is a dynamic quenching process with two binding modes: a strong one under low Ag^+^ concentration and a weak one under high Ag^+^ concentration [57].

**Fig. 3:**
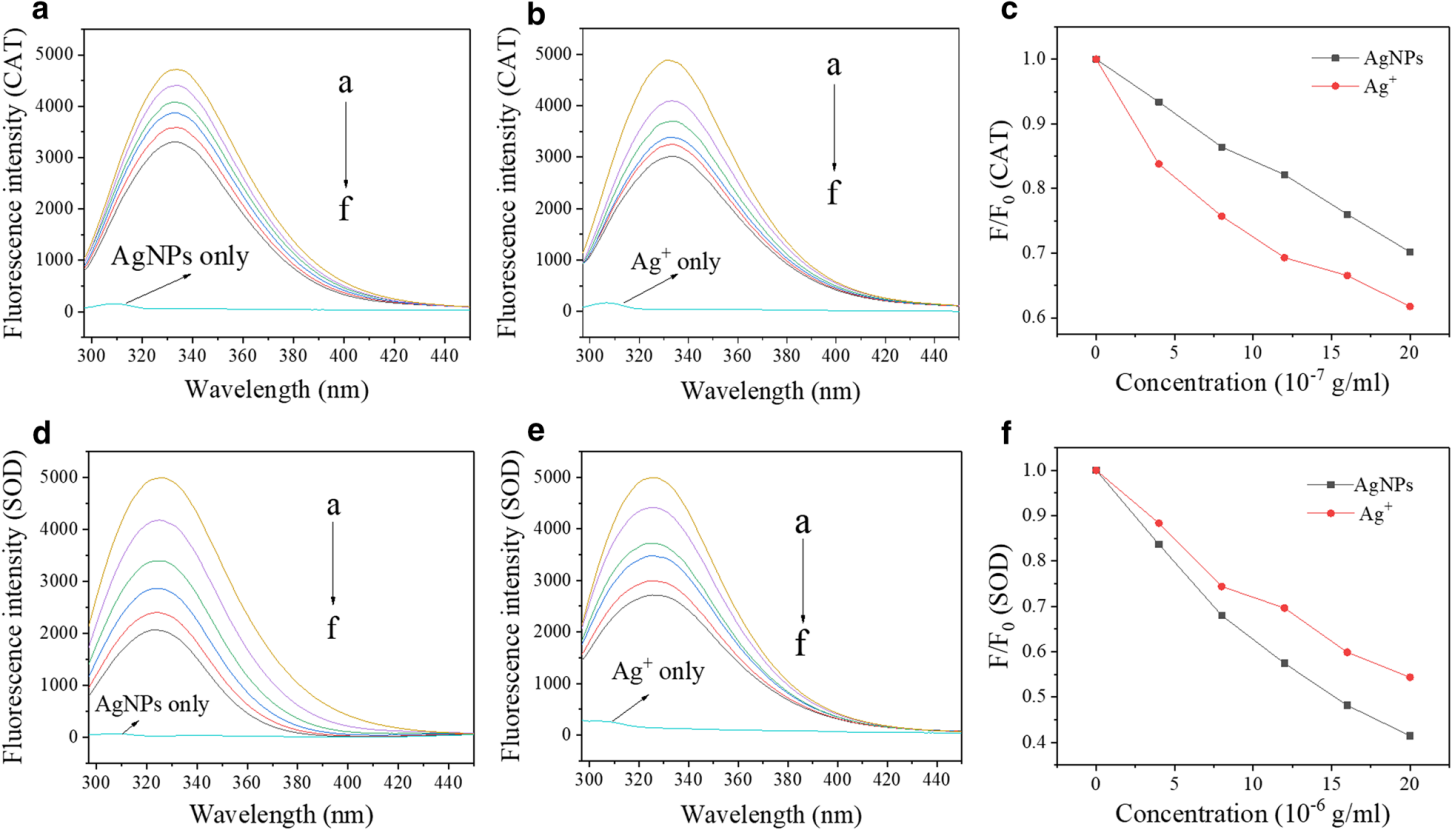
Fluorescence spectra of CAT (**a**, **b**) or SOD (**d**, **e**) in the presence of AgNPs (**a**, **d**) or Ag^+^ (**b**, **e**) at different concentrations; F/F_0_ (Spectral peak) of CAT (**c**) or SOD (**f**) in the presence of AgNPs or Ag^+^ at different concentrations. (Where F_0_ is the fluorescence intensity of CAT/SOD without quencher, F is the fluorescence intensity of CAT/SOD quenched by AgNPs/Ag^+^, the same as below). Condition: T = 298 K; pH 7.4; **a**–**c** $\lambda_{ex}$ = 278 nm; C(CAT) = 5×10^−7^ mol/L; C(AgNPs/Ag^+^, × 10^−7^ g/mL), a–f 0, 4, 8, 12, 16, 20; AgNPs or Ag^+^ only: C(CAT) = 0, C(AgNPs or Ag^+^) = 20 × 10^−7^ g/mL; **d**–**f** $\lambda_{ex}$ = 280 nm; C(SOD) = 6×10^−6^ mol/L; C(AgNPs/Ag^+^, × 10^−6^ g/mL), a–f 0, 4, 8, 12, 16, 20; AgNPs or Ag^+^ only: C(SOD) = 0, C(AgNPs or Ag^+^) = 20 × 10^−6^ g/mL

The intrinsic fluorescence spectra of CAT/SOD under different concentrations of AgNPs and Ag^+^ are shown in Fig. 3. The addition of AgNPs or Ag^+^ did not significantly change the peak position of CAT fluorescence spectra. However, with the increase in the concentrations of AgNPs and Ag^+^, the fluorescence intensity of CAT decreased regularly, indicating that they had a quenching effect on the endogenous fluorescence of CAT.

AgNPs caused a slight blue shift in the peak position of SOD fluorescence emission spectrum, indicating that the increased hydrophobicity of fluorescent groups and the decreased polarity. It was speculated that the polarity of the main source of endogenous fluorescence (tryptophan and tyrosine residues) was changed. The addition of Ag^+^ did not lead to the significant peak shift of SOD. With the increase in the concentrations of AgNPs and Ag^+^, both of them had the quenching effect on the endogenous fluorescence of SOD. The quenching effect of Ag^+^ on the intrinsic fluorescence of CAT was stronger than that of AgNPs, whereas the quenching effect of AgNPs on the intrinsic fluorescence of SOD was stronger than that of Ag^+^.

Compared with AgNPs, Ag^+^ could be bound to CAT with higher affinity. Therefore, the decrease in CAT activity caused by the increase in Ag^+^ concentration was more significant than that caused by the increase in AgNPs concentration. Compared with Ag^+^, AgNPs could be bound to SOD with higher affinity. The difference was the reason that the increase in AgNPs concentration led to the decrease in SOD activity, whereas the increase in Ag^+^ concentration enhanced the SOD activity, when AgNPs or Ag^+^ was directly applied on enzymatic proteins.

### Influences of AgNPs and Ag^+^ on the microenvironment of CAT

**Fig. 4:**
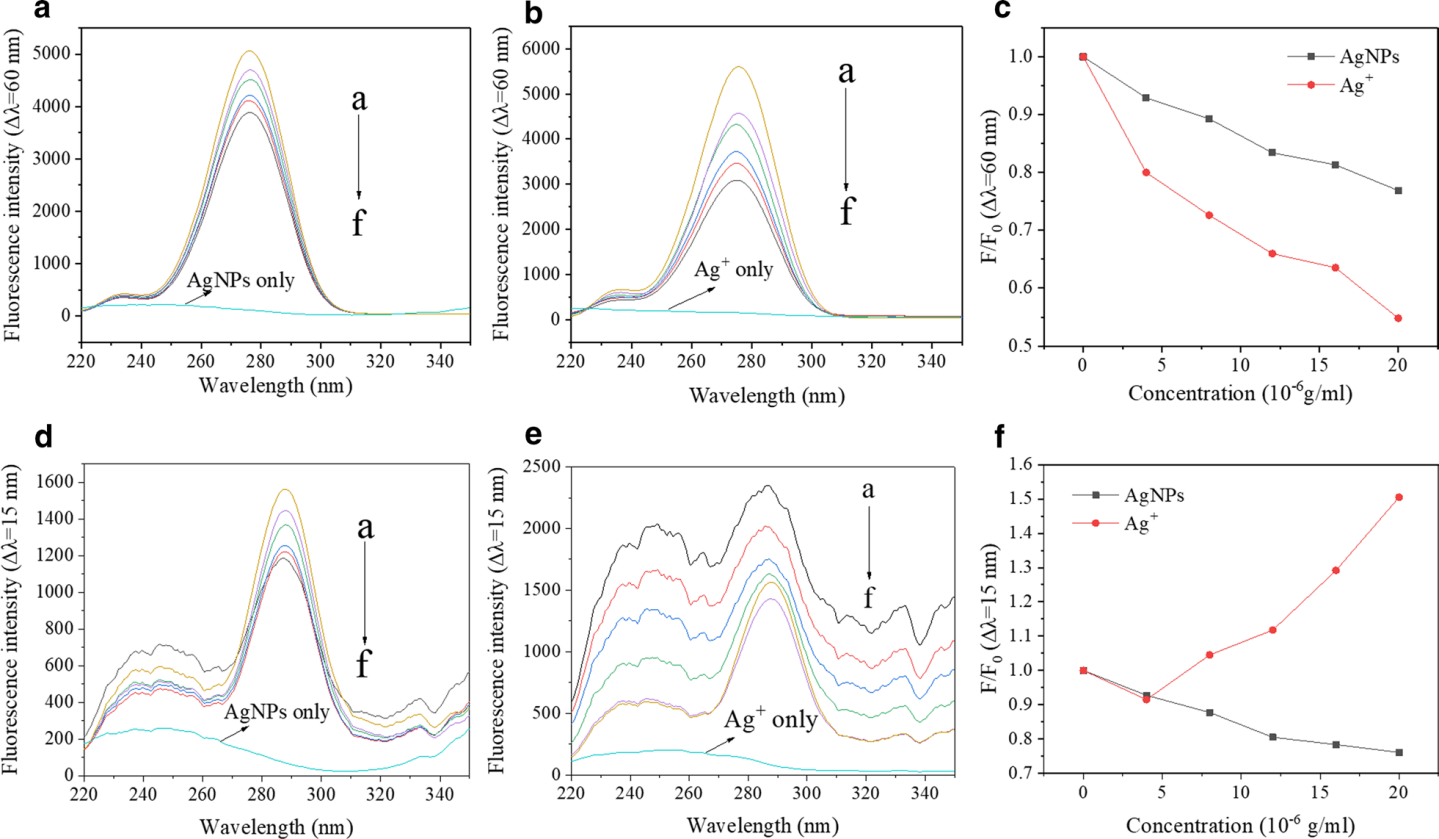
Synchronous fluorescence spectra of CAT in the presence of AgNPs (**a**, **d**) or Ag^+^ (**b**, **e**) at different concentrations. F/F_0_ (Synchronous fluorescence spectral peak) of CAT in the presence of AgNPs or Ag^+^ at different concentrations. Condition: T = 298 K; pH 7.4; C(CAT) = 5×10^−7^ mol/L; AgNPs (**a**)/Ag^+^ (**b**) only: C(CAT) = 0, C(AgNPs/Ag^+^) = 20 × 10^−7^ g/mL; **a**, **b**, **d** C(AgNPs/Ag^+^, × 10^−7^ g/mL), a–f 0, 4, 8, 12, 16, 20; **e** C(Ag^+^, × 10^−7^ g/mL), a–f 20, 16, 12, 8, 0, 4

The synchronous spectra obtained at $\Delta\uplambda$ = 15 nm showed the spectral characteristics of Tyr, whereas the synchronous fluorescence spectra at $\Delta\uplambda$ = 60 nm showed the spectral characteristics of Trp [58, 59]. Synchronous fluorescence spectra can be used to investigate the effects of molecules on protein conformation and detect the microenvironment of endogenous fluorescent amino acid residues in proteins [60]. With the increase in the concentration of AgNPs or Ag^+^, the fluorescence intensity of Trp was decreased (Fig. 4a–c). At the same time, there was no obvious red shift or blue shift in the position of fluorescence emission peaks, indicating that neither AgNPs nor Ag^+^ could significantly change the micro-environment of CAT tryptophan residues.

Figure 4d shows the changes in the microenvironment of tyrosine residues caused by the addition of AgNPs. With the increase in AgNPs concentration, the position of the fluorescence emission peak of tyrosine residues showed a slight blue shift, indicating that AgNPs slightly changed the CAT tyrosine residue microenvironment and enhanced the hydrophobicity.

Figure 4e shows the changes in the microenvironment of tyrosine residues caused by the addition of Ag^+^. With the increase in Ag^+^ concentration, the fluorescence intensity firstly decreased and then increased. It was reported that Ag^+^ could react with 6-mercaptopurine to form highly fluorescent complexes under similar conditions (pH 7.2) [61]. Also the existing literature indicated that tryptophan could interact with gold nanorods and lead to enhancement in fluorescence intensity [62]. It was speculated that the increase in the fluorescence intensity was ascribed to highly fluorescent complexes generated in the reaction between high concentration of Ag^+^ and CAT. The reaction made protein molecules contract, thus enhancing energy transfer among amino acids as well as the fluorescence intensity [63]. The fluorescence emission peaks of CAT tyrosine residues were slightly redshifted. The addition of Ag^+^ weakened the hydrophobicity and enhanced the polarity of CAT tyrosine residues.

Although the influences of Ag^+^ and AgNPs on CAT were different (positive effect or negative effect), compared with AgNPs, Ag^+^ had a stronger effect on the fluorescence change in tryptophan residues and tyrosine residues of CAT, thus leading to the stronger inhibition of Ag^+^ on enzyme activity at higher Ag^+^ concentrations.

### Influences of AgNPs and Ag^+^ on the microenvironment of SOD

**Fig. 5:**
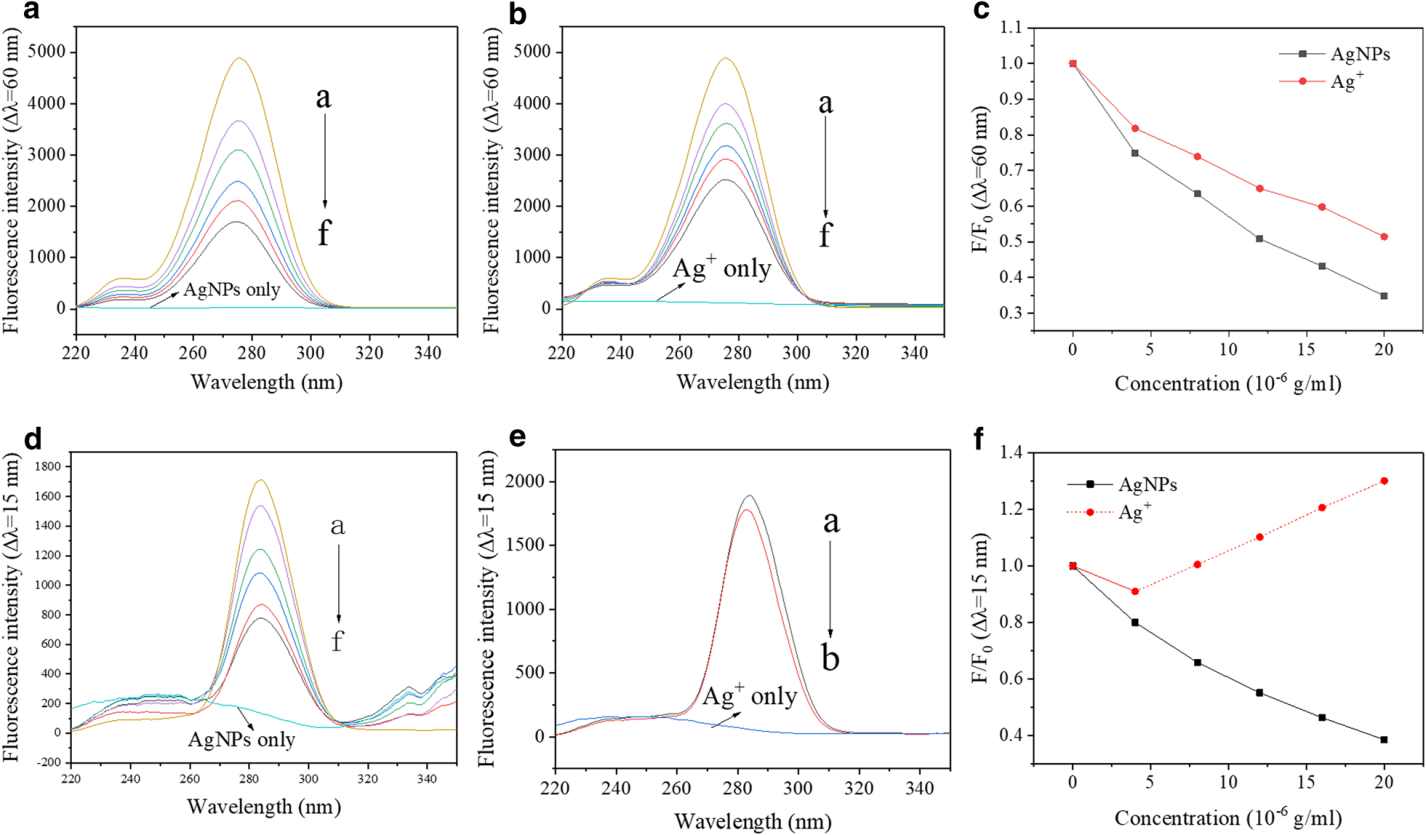
Synchronous fluorescence spectra of SOD in the presence of AgNPs (**a**, **d**) or Ag^+^ (**b**, **e**) at different concentrations; F/F_0_ (Synchronous fluorescence spectral peak) of SOD in the presence of AgNPs or Ag^+^ at different concentrations. Condition: T = 298 K; pH 7.4; C(SOD) = 6×10^−6^ mol/L. **a**, **b**, **d** C(AgNPs/Ag^+^, × 10^−6^ g/mL), a–f: 0, 4, 8, 12, 16, 20; AgNPs/Ag^+^ only: C(SOD) = 0, C(AgNPs/Ag^+^)=20 × 10^−6^ g/mL; **e** C(AgNPs/Ag^+^, × 10^−6^ g/mL), a–b: 0, 4; Ag^+^ only: C(SOD) = 0, C(Ag^+^)=4 × 10^−6^ g/mL

The addition of AgNPs resulted in a slight blue shift in the position of SOD fluorescence peak (Fig. 5a–c), indicating that AgNPs enhanced the hydrophobicity and weakened the polarity of SOD tryptophan residues in the microenvironment. Ag^+^ did not cause a significant shift in the peak position, indicating that Ag^+^ did not cause a significant change in the microenvironment polarity of tryptophan residues.

With the increase in AgNPs concentration, the position of the fluorescence emission peak of the tyrosine residues did not move obviously (Fig. 5d), indicating that AgNPs did not significantly change the tyrosine residue microenvironment. It was found that with the increase in Ag^+^ concentration, the position of the fluorescence emission peak was blue-shifted, indicating that Ag^+^ could change the conformation of SOD, thus increasing the hydrophobicity of the SOD tyrosine residue microenvironment. The fluorescence intensity of SOD tyrosine residues decreased with the addition of the low concentration of Ag^+^ (Fig. 5e). When the concentration of Ag^+^ further increased to 8 × 10^−6^ g/mL, the same trend to CAT activity was observed (the dotted line in Fig. 5f). The fluorescence intensity firstly decreased and then increased with the increase in Ag^+^ concentration. The result might be ascribed to the formation of highly fluorescent complexes [61] between Ag^+^ and SOD and the binding site was tyrosine residues of SOD.

Synchronous fluorescence spectra showed that the quenching effect of AgNPs on tryptophan residue fluorescence of SOD was stronger than that of Ag^+^. Moreover, Ag^+^ did not change the microenvironment of tryptophan residues, whereas AgNPs enhanced the hydrophobicity and weakened the polarity of SOD tryptophan residues. Under a low concentration (0–4 × 10^−6^ g/mL), the effect of AgNPs on the fluorescence quenching of SOD tyrosine residues was stronger than that of Ag^+^. These results were consistent with the inhibition of SOD activity caused by a high concentration of AgNPs.

### Aggregation state of AgNPs and Ag^+^ with CAT/SOD

**Fig. 6:**
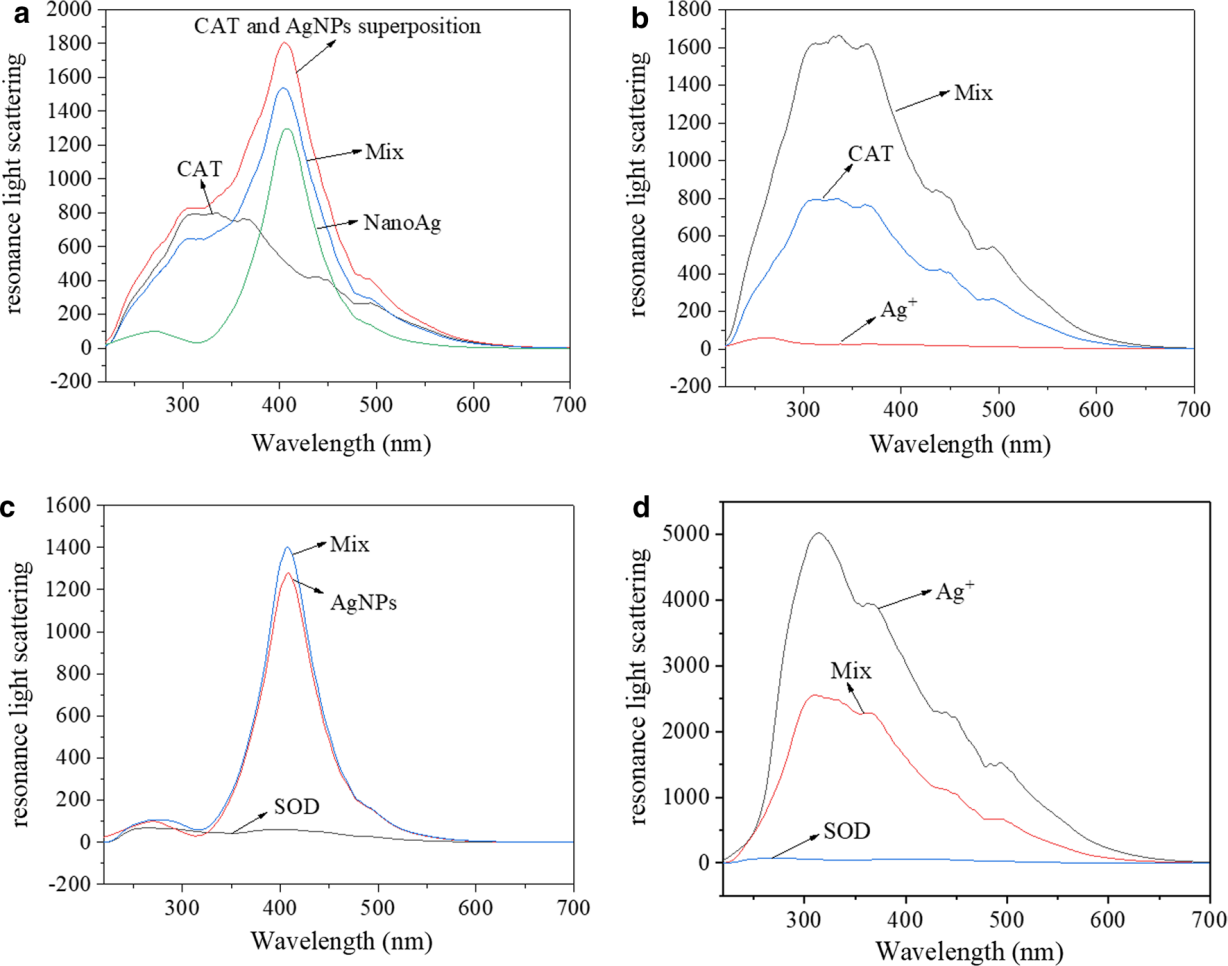
Resonance light scattering spectra of AgNPs (**a**, **c**)/Ag^+^ (**b**, **d**) and/or CAT (**a**, **b**)/SOD (**c**, **d**). Condition: T = 298 K; pH 7.4; $\lambda_{ex}$ = $\lambda_{em}$ = 220 nm; **a**, **b** Mix: C(CAT) = 5×10^−7^ mol/L, C(AgNPs/Ag^+^) = 8 × 10^−7^ g/mL; CAT: C(CAT) = 5×10^−7^ mol/L; AgNPs/Ag^+^: C(AgNPs/Ag^+^) = 8 × 10^−7^ g/mL; **c**, **d** Mix: C(SOD) = 6 × 10^−6^ mol/L, C(AgNPs/Ag^+^) = 8 × 10^−6^ g/mL; SOD: C(SOD) = 6×10^−6^ mol/L; AgNPs/Ag^+^: C(AgNPs/Ag^+^) = 8 × 10^−6^ g/mL

RLS experiments are usually used to explore the aggregation state between materials. For aggregated species, enhanced light scattering can be observed in the wavelength characteristics of these species [39] The RLS of CAT near 350 nm was strong, whereas the intensity of Ag^+^ peak near 350 nm was weak under the experimental conditions (Fig. 6a). The RLS intensity of CAT–Ag^+^ was higher than that of CAT and Ag^+^, indicating that the formation of CAT–AgNPs aggregates increased the average particle diameter. The RLS intensity of CAT–AgNPs mixed system was lower than that of AgNPs or CAT (Fig. 6b). The results showed that polymerization did not occur between AgNPs and CAT and that the CAT–AgNPs system became more uniform without increasing the particle size.

At 314 nm, the RLS intensity of Ag^+^ was strong and the intensity of SOD was weak. The RLS intensity of SOD–Ag^+^ was significantly lower than that of Ag^+^, indicating that the SOD–Ag^+^ system became more uniform without increasing the particle size. The RLS intensity of AgNPs was strong and the RLS intensity of SOD at 408 nm was weak. The RLS intensity of AgNPs–SOD was higher than that of AgNPs, indicating that polymerization occurred between AgNPs and SOD and increased the average particle diameter.

The results of resonance light scattering studies are shown in Table 1. The above results were consistent with the difference that Ag^+^ (AgNPs) had the stronger inhibitory effect on CAT (SOD) activity than AgNPs (Ag^+^), indicating that the aggregation state and dispersion size were two key factors influencing the activity of CAT/SOD during the direct interaction between molecules.

**Table 1:** Aggregation state of AgNPs and Ag^+^ with CAT/SOD

	CAT	SOD
AgNPs	More homogenous	Occurred polymerization
Ag^+^	Occurred polymerization	More homogenous

### Influences of AgNPs and Ag^+^ on protein structures of CAT/SOD

**Fig. 7:**
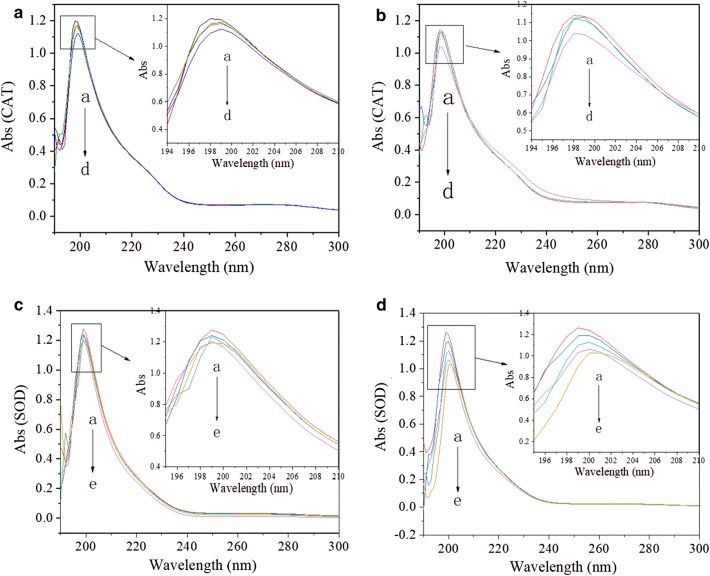
The absorption spectra of CAT (**a**, **b**) and SOD (**c**, **d**) in the presence of AgNPs (**a**, **c**) or Ag^+^ (**b**, **d**) at different concentrations. Condition: T = 298 K; pH 7.4; **a**, **b** C(CAT) = 5×10^−7^ mol/L; C(AgNPs/Ag^+^, × 10^−6^ g/mL), a–d: 0.2, 0.4, 0.6, 0.8; **c**, **d** C(SOD) = 5×10^−7^ mol/L; C(AgNPs/Ag^+^, × 10^−6^ g/mL), a–e: 2, 4, 6, 8, 10

Most proteins have two absorption peaks at 205 nm and 280 nm respectively corresponding to peptide skeleton and the aromatic ring amino acids [64–66]. With the increase in AgNPs or Ag^+^ concentration, the skeleton absorption peak of CAT and SOD decreased (Fig. 7). The results showed that both AgNPs and Ag^+^ changed the protein skeleton of enzyme molecules, thus loosening the skeleton structure, unfolding the polypeptide chain, and gradually exposing the amino acids in the molecules.

The UV absorption spectra showed that the influences of Ag^+^ on the skeleton structures of CAT and SOD were more significant than those of AgNPs (Fig. 7). These results indicated that the effect of AgNPs or Ag^+^ on the protein skeleton was also one of the reasons for the change in CAT/SOD activity in the direct interaction between molecules.

## Conclusions

In this work, we explored the toxicity of silver nanoparticles (20 nm) and silver ions to RBCs and antioxidant enzymes (CAT, SOD and GPX).

AgNPs and Ag^+^ could affect the enzymatic and non-enzymatic antioxidant system of RBCs. CAT, SOD and GPX were more sensitive to Ag^+^, whereas the RBCs had slightly higher GSH contents after the treatment with AgNPs. Both AgNPs and Ag^+^ increased the MDA content of RBCs, but the difference between the effects of AgNPs and Ag^+^ was not significant. We speculated that multiple factors, including the cell membrane penetration ability of AgNPs and Ag^+^, led to the results. The difference in the change in the enzyme activity indicated that AgNPs and Ag^+^ might have different influencing mechanisms on CAT and GPX. And SOD has stronger resistance to both of AgNPs and Ag^+^.

The direct interaction among molecules (AgNPs/Ag^+^ and CAT/SOD) was one of the important reasons for the change in the antioxidant enzyme activity in the exposure at cell level. The spectral analysis results showed that the interaction mechanism and conformational changes were also the important factors for the changes in the enzyme activity when enzyme proteins at the molecular level were directly exposed to the AgNPs or Ag^+^.

## Data Availability

The datasets used and/or analysed during the current study are available from the corresponding author on reason able request.

## Abbreviations

RBCs: red blood cells
CAT: catalase
AgNPs: silver nanoparticles
SOD: superoxide dismutase
GPX: glutathione peroxidase
GSH: glutathione
MDA: malondialdehyde
ROS: reactive oxygen species
SD: standard deviation
PBS: phosphate-buffered saline
RLS: resonance light scattering
Trp: tryptophan
Tyr: tyrosine
Phe: phenylalanine
